# Dataset on the interview-based survey of Moscow bicycle infrastructure

**DOI:** 10.1016/j.dib.2019.104429

**Published:** 2019-08-24

**Authors:** Nadezhda Zavyalova, Vladimir Kolmakov, Aleksandra Polyakova, Dmitriy Zavyalov

**Affiliations:** a Plekhanov Russian University of Economics, 36 Stremyanny Lane, Moscow, 117997, Russia; b Industrial University of Tyumen, 38 Volodarskogo Street, Tyumen, 625000, Russia

**Keywords:** Urban transit system, Cycling infrastructure, Means of transport, Public survey, Computer assisted telephone interview, Urban planning

## Abstract

The data were collected using Computer Assisted Telephone Interview with a questionnaire set of closed-ended and open-ended questions. Respondents’ phone numbers were randomly generated. The survey sample included 1000 adult citizens of Moscow – active and potential users of bicycles (in equal proportions) who take or can take regular rides to destinations up to 5 km away, as of August 2018. The sample was representative in terms of gender-age and geographical distribution characteristics of the population.

The data table is supplemented with the original questionnaire. Survey results were put in the database containing respondents’ metadata and their answers, either encoded or provided as a plain text (for open-ended questions). No other processing was applied to the data to maintain suitability for different analyses.

The data can be used in further research of cities’ public transport infrastructure to evaluate its performance and returns on investment, to formulate initial hypotheses, to draft a survey strategy and design, and to verify results, etc. The provided questionnaire is a ready-to-deploy instrument for custom surveys in the related areas of research.

**Table:** Specifications Table

Subject	Geography, Planning and Development
Specific subject area	Urban transit system development, cycling infrastructure design concerning the individuals' preferences of bicycle use
Type of data	Table supplemented with a questionnaire provided as supplementary file
How data were acquired	Computer Assisted Telephone Interview
Data format	Raw
Parameters for data collection	The survey sample included 1000 adult (18+) citizens of Moscow – active and potential users of bicycles (in equal proportions) who take or can take regular rides to destinations up to 5 km away, as of August 2018. The sample was representative in terms of gender-age and geographical distribution characteristics of the population.
Description of data collection	The data were collected using Computer Assisted Telephone Interview, numbers randomly generated. Matters of interest included: – Bicycle traffic situation in Moscow: cyclists' share in the population and its growth potential, barriers and incentives; – Cyclists' needs and behaviour patterns: ride frequency, routes and destinations, duration, a will to use a bicycle as a regular transport means; – Individuals' assessment of the city efforts to develop cycling infrastructure: demanded vectors of development, overall quality, willingness to support.
Data source location	Moscow, Russia
Data accessibility	With the article

**Table:** 

**Value of the data** • The data can be used to analyse individuals' attitudes towards city transit system development in Moscow and other comparable cities having the same population, planning features and transport situation. • The main beneficiaries of the data include urban studies researchers, transportation systems specialists, sociologists and marketing specialists. • The data can be used in further research to formulate the initial hypothesis, to draft a survey strategy and design, and to verify results, etc. • The provided questionnaire is a ready-to-deploy instrument for the same surveys in any other city or for custom surveys in the related areas of research, as well as the provided data can serve as the valid control sample for proper verification. • Deeper analysis of the data can be valuable for different valuations of transport infrastructure performance or social return on investment in the infrastructure in Moscow or in other alike cities, of the perspectives of economic cluster formation [1] around the cycling and sharing infrastructure.

## 1 Data

The dataset in this article describes the results of interview-based survey of Moscow bicycle infrastructure. Table 1 contains gender-age characteristics of the sample. Table 2 provides the sample territorial breakdown with the population control-set. The “Data” tab of the supplementary Microsoft Excel worksheet contains 1000 entries of respondents’ answers to the questions listed in the “Questions encoded” tab. Answers are either encoded or present as plain text.

**Table 1:** The sample gender-age distribution.

Age cohort	Women	Men	Total
18-29 y. o.	182	253	435
30-39 y. o.	125	172	297
40-49 y. o.	58	94	152
50-59 y. o.	30	46	76
60 y. o. and older	13	27	40
Total	408	592	1000

The sample's territory breakdown was not controlled, yet the population territorial distribution proportions were followed as of January 2018 (see Table 2 for details).

**Table 2:** The sample territorial breakdown with the population control-set.

Territory	Population^a^		Sample		Deviation, perc. points
	People	%	People	%	
Moscow City total	12506468	100.0	1000	100.0	–
Eastern Administrative Okrug	1515942	12.1	141	14.1	−2.0
Western Administrative Okrug	1382516	11.1	83	8.3	2.8
Zelenogradskiy Administrative Okrug	243084	1.9	20	2.0	−0.1
Novomoskovskiy - Troitskiy Administrative Okrug	358897	2.9	26	2.6	0.3
Northern Administrative Okrug	1176611	9.4	113	11.3	−1.9
North-eastern Administrative Okrug	1423956	11.4	112	11.2	0.2
North-western Administrative Okrug	1001346	8.0	69	6.9	1.1
Central Administrative Okrug	775881	6.2	80	8.0	−1.8
South-eastern Administrative Okrug	1405650	11.2	100	10.0	1.2
South-western Administrative Okrug	1437242	11.5	117	11.7	−0.2
Southern Administrative Okrug	1785343	14.3	139	13.9	0.4

Territorial breakdown deviations of the sample are insignificant and can be neglected since they cannot mislead the research outcome.

a Otsenka chislennosti postoyannogo naseleniya g. Moskvy na 1 yanvarya 2018 g. [Moscow permanent population estimate for the 1st of January 2018] (in Russian)/Moscow Statistics Authority. URL: http://moscow.gks.ru/wps/wcm/connect/rosstat_ts/moscow/ru/statistics/population/(accessed 15 July 2019).

The data is supplemented by the questionnaire (see the appropriate file). Data interpretation comments upon the encoded answers are available in the questionnaire and/or the data file. Original questionnaire and worksheet (respondents’ answers to open-ended questions) are in Russian. English translation was made by the authors. Interviewer name, respondent name (question A1) and phone number are excluded from the data to maintain privacy.

## 2 Experimental design, materials, and methods

The data were collected in August 2018 during a survey of bicycle traffic development in Moscow. The data were acquired using the Computer Assisted Telephone Interview with phone numbers randomly generated.

The assessed characteristics of sample included the following:

– cyclists' share in the population and its growth potential,
– barriers and incentives for using a bicycle as an every-day transport means;
– cyclists' needs and behaviour patterns: frequency of rides, routes and destinations, duration of rides, etc.;
– individuals' assessment of the city efforts to develop cycling infrastructure: overall evaluation of quality, knowledge of the city's infrastructure projects being implemented, the demanded vectors of the infrastructure development.

The structured questionnaire used for the data collection followed Malhorta's basic requirements and guidelines [2] to questionnaire design and contained the below mentioned sections:

1. Screening questions to filter and verify quotas:
  – territory of living;
  – means of transport used to travel around the city;
  – frequency of a bicycle use;
  – average bicycle ride duration, etc.
2. Main section of questions aimed at assessment of an individual's perception of bicycle infrastructure development in terms of “Safety”, “Barrier free environment”, “Connectedness”, “Attractiveness”, “Comfort”.

The survey sample included 1000 adult (18+) citizens of Moscow – active and potential users of bicycles (in equal proportions) who take or can take regular rides to destinations up to 5 km away, as of August 2018. The sample was representative in terms of gender-age and geographical distribution characteristics of the population. The sample size determination procedure followed the guidelines described by R. Lenth [3] in line with methodology background provided by Adcock [4]. For the population size of 12.506 million people^1^ the two groups of respondents (active and potential cyclists) had to be represented by 720 people each to achieve 95% confidence level with 5% error margin for the two-parametric survey. Given the number of parameters in scope was much higher, we followed Dell, Holleran & Ramakrishnan's [5] proposition that a sample size was not sensitive to population change over 20000, thus absorbing the multiple parameters in the population. To meet the minimum requirement of the sample representation (1440 people) we questioned 1000 more people in the street using the same questionnaire. The “street” sample was used for control and validation purposes, it is not included in the data.

Age and gender were the two parameters to control the sample matching the population. Actual gender distribution within the sample was 59.2% men and 40.8% women while the population structure in January 2018 was 46.2% and 53.8% respectively. Age distribution within the sample drifted towards younger cohorts (18–49 y. o., 89% of the sample), that matched our expectations about the characteristics of potential bicycle users. See Table 1 for the sample gender-age distribution.

## Footnotes

1 Raspredelenie postoyannogo naseleniya po polu i vozrastu [Permanent population gender and age distribution] (in Russian)/Moscow Statistics Authority. URL: http://moscow.gks.ru/wps/wcm/connect/rosstat_ts/moscow/ru/statistics/population/(accessed 15 July 2019).
