# Supplementary material for: Dataset on the interview-based survey of Moscow bicycle infrastructure
Source: Data Brief. 2019 Aug 24;26:104429. doi: 10.1016/j.dib.2019.104429 (PMC6734097; doi:10.1016/j.dib.2019.104429)
Supplement: Multimedia component 2 [file mmc2.pdf]

Cycling Infrastructure Monitoring

THE BEGINNING

Questionnaire number

Hello! We kindly ask you to participate in the survey.

We study residents' satisfaction with the development of pedestrian and cycling infrastructure for the benefit of people in Moscow. The survey takes up to 10 minutes to complete. The questionnaire is anonymous, and the data will be summarized.

A1. What is your name?

INT. PLEASE WRITE IN ______________

A2. Respondent's sex

INT. SPECIFY IT AUTOMATICALLY

QUOTAS BY PARAMETER

1. Male
2. Female

A3. How old are you? (age at last birthday)

INT. DO NO READ OUT, SPECIFY IT BASED ON THE RESPONDENT'S ANSWER. ONE ANSWER

QUOTAS BY PARAMETER

1. Less than 18
2. 18-29
3. 30-39
4. 40-49
5. 50-59
6. 60+

A4. Which administrative okrug do you live in?

INT. DO NO READ OUT, SPECIFY IT BASED ON THE RESPONDENT'S ANSWER. ONE ANSWER

QUOTAS BY PARAMETER

1. Eastern Administrative Okrug
2. Western Administrative Okrug
3. Northern Administrative Okrug
4. Northeastern Administrative Okrug
5. Northwestern Administrative Okrug
6. Central Administrative Okrug
7. Southeastern Administrative Okrug
8. South-Western Administrative Okrug
9. Southern Administrative Okrug
10. Zelenogradskiy Administrative Okrug
11. Novomoskovskiy-Troitskiy Administrative Okrug
12. Moscow Oblast
13. Other END INTERVIEW

A4.1. Which district of Moscow do you live in?
ENCODE IT. ONE ANSWER.

Arbat	Vykhino-Zhulebino	Kryukovo	Nagatinskiy Zaton	Ryazanskiy	Troparevo-Nikulino
Academicheskiy	Gagarinskiy	Kuzminki	Nagorny	Savelki	Filevskiy Park
Alekseevskiy	Golovinskiy	Kuntsevo	Nekrasovka	Savelovskiy	Fili Davydkovo
Altufevskiy	Golyanovo	Kurkino	Nizhegorodskiy	Sviblovo	Khamovniki
Aeroport	Danilovskiy	Levoberezhny	Novogireevo	Severnoe Butovo	Khovrino
Babushkinskiy	Dmitrovskiy	Lefortovo	Novokosino	Severnoe Izmailovo	Khoroshevo-Mnevniki
Basmanny	Donskoy	Lianozovo	Novo-Peredelkino	Severnoye Medvedkovo	Khoroshevskiy
Begovoy	Dorogomilovo	Lomonosovskiy	Obruchevskiy	Severnoe Tushino	Tsaritsyno
Beskudnikovskiy	Zamoskvorechye	Losinoostrovskiy	Orekhovo-Borisovo Severnoe	Severny	Cheryomushki
Bibirevo	Zapadnoe Degunino	Lyublino	Orekhovo-Borisovo Yuzhnoe	Silino	Chertanovo Severnoe
Biryulyovo Vostochnoe	Zyuzino	Marfino	Ostankino	Sokol	Chertanovo Centralnoe
Biryulyovo Zapadnoe	Zyablikovo	Maryina Roshcha	Otradnoe	Sokolinaya Gora	Chertanovo Yuzhnoe
Bogorodskoe	Ivanovo	Maryino	Ochakovo-Matveyevskoe	Sokolniki	Schukino
Brateevo	Izmailovo	Matushkino	Perovo	Solntsevo	Yuzhnoe Butovo
Butyrsky	Kapotnya	Metrogorodok	Pechatniki	St. Kryukovo	Yuzhnoye Medvedkovo
Veshnyaki	Konkovo	Meshchansky	Pokrovskoe Streshnevo	Strogino	Yuzhnoe Tushino
Vnukovo	Koptevo	Mitino	Preobrazhenskoe	Taganskiy	Yuzhnoportovy
Voikovskiy	Kosino-Ukhtomskiy	Mozhayskiy	Presnenskiy	Tverskoy	Yakimanka
Vostochnoe Degunino	Kotlovka	Molzhaninovskiy	Prospekt Vernadskogo	Tekstilschiki	Yaroslavskiy
Vosochnoe Izmailovo	Krasnoselskiy	Moskvorechye-Saburovo	Ramenki	Teply Stan	Yasenevo
Vostochny	Krylatskoe	Nagatino-Sadovniki	Rostokino	Timiryazevskiy	

**Novomoskovskiy Adninistrative Okrug**	**Troitskiy Administrative Okrug**
Vnukovskoe	Voronovskoe
Voskresenskoe	Kievskiy
Desyonovskoe	Klyonovskoe
Kokoshkino	Krasnopahorskoe
Marushkinskoe	Mikhailovo-Yartsevskoe
Moscovskiy	Novofedorovskoe
Mosrentgen	Pervomayskoe
Ryazanovskoe	Rogovskoye
Sosenskoe	Troitsk
Filimonkovskoe	Shchapovskoe
Shcherbinka	

98. Refusal to answer END INTERVIEW
99. Not sure END INTERVIEW

A5. Do you ride a bicycle in parks, in the city, etc.?

INT. READ OUT, ONE ANSWER

1. Yes
2. No, I know how to ride a bicycle, but I don't cycle GO TO QUESTION PA3
3. No, I don't know how to ride a bicycle END INTERVIEW

A 5.1. Over the past year or two years, have you started to cycle more or less often? Or is there no change in the frequency? READ OUT THE OPTIONS, ONE ANSWER

1. I've started to cycle more often
2. I've started to cycle less often
3. No change
4. DO NOT READ OUT. Not sure

IF THERE ARE CODES 1 OR 2 IN A 5.1

A6. Where do you cycle more often?

INT. READ OUT, SEVERAL OPTIONS CAN BE CHOSEN

1. Leisure activity
2. Work
3. Educational institution (university, etc.)
4. Shops, hospitals, banks, etc.
5. Metro stations, public transport stops, train station
6. Meeting with friends, café, social events
7. Sports
8. Other __________ INT. WRITE IN THE ANSWER
9. DO NOT READ. Not sure

ASK IF THERE IS CODE 1 IN A5

A7. How often do you ride a bicycle in the warm season (from April to October)?

INT. DO NOT READ OUT, SPECIFY IT BASED ON THE RESPONDENT'S ANSWER. ONE ANSWER

1. Every day
2. Several times a week
3. Once a week
4. Several times a month
5. Once a month or less often

ASK IF THERE ARE CODES 4 AND 5 IN A7

A8. Are you ready to ride a bicycle more often in the city?

Use 10-point scale, where 10 means "Certainly ready", 1 means "Not ready at all".

Not ready at all									Certainly ready
1	2	3	4	5	6	7	8	9	10

99 – not sure

ASK IF THERE IS CODE 1 IN A5

A9. How long is your average bike ride?

INT. READ OUT. ONE ANSWER PER EACH LINE

WHEN ENCODING, LINK IT WITH THE PURPOSE (A6)

	**1. LEISURE** (ask if there is code 1 in A6)	**2.BUSINESS** (ask if there are codes 2-6 in A6)	**3.SPORT** (ask if there is code 7 in A6)
Up to 15 min			
15 - 30 min			
30 min – 1 hour			
Over 1 hour			
DO NOT READ OUT<br>Not sure/ No answer			

A10. How satisfied are you with quality of the Moscow cycling infrastructure in general?

(bike paths, road signs and display boards, bicycle parking, bike rentals, etc.)

INT. SPECIFY IT BASED ON THE RESPONDENT'S ANSWER. ONE ANSWER

Use 10-point scale, where 10 means "Fully satisfied", 1 – "Not satisfied at all".

You can use any point from 1 to 10 to express your opinion in the best way.

Not satisfied at all									Fully satisfied
1	2	3	4	5	6	7	8	9	10

99 – not sure

FURTHER, THOSE WHO STATED CODE 1 IN A5 GO TO BLOCK B

THOSE WHO STATED CODE 2 – BLOCK P

BA1. Do you ride your own or rental bicycle?

INT. READ OUT, ONE ANSWER

1. Only my own bicycle
2. Only rental bicycle
3. Both my own and rental bicycles, it depends on circumstances

ASK IF THERE IS CODE 1 IN A5

BA2. What other modes of transport do you use to move around the city at least once a week?

INT. READ OUT, SEVERAL OPTIONS CAN BE CHOSEN

1. Public transport
2. Private car
3. Taxi
4. Motorcycle
5. Carsharing
6. Scooter, skateboard, etc.
7. Other ___________ INT. WRITE IN THE ANSWER

TRAFFIC SAFETY

Now, let's talk about bicycle safety in Moscow.

BB1. Based on your experience of cycling in Moscow, please assess bicycle safety by the following characteristics:

INT. READ OUT THE SCALE IF NECESSARY

Use 10-point scale, where 10 means "Fully safe", 1 – "Not safe at all".

INT. ONE ANSWER PER EACH LINE

IF A RESPONDENT IS NOT SURE ABOUT HIS OR HER ANSWER, SPECIFY 99 – NOT SURE

ADD ROTATION

Question No.	Characteristic	Scale
1.1	Cycling in crosswalks	1 2 3 4 5 6 7 8 9 10<br>99
1.2	Cycling in underground crosswalks	1 2 3 4 5 6 7 8 9 10<br>99
1.3	Cycling on public transport lanes	1 2 3 4 5 6 7 8 9 10<br>99
1.4	Road cycling	1 2 3 4 5 6 7 8 9 10<br>99
1.5	Cycling on bike paths	1 2 3 4 5 6 7 8 9 10<br>99
1.6	Cycling on bike lanes on roadways	1 2 3 4 5 6 7 8 9 10<br>99
1.7	Cycling in pedestrian zones without bike paths	1 2 3 4 5 6 7 8 9 10<br>99
1.8	Cycling in residential areas	1 2 3 4 5 6 7 8 9 10<br>99

BB2. Where does a cyclist have to ride if there is no bike path, in accordance with traffic rules?

INT. READ OUT. ONE ANSWER

1. On the sidewalk
2. On the roadway
3. Both options are acceptable
4. DO NOT READ OUT Not sure

BB3. How often do you ride on bike lanes on roadways during your typical ride?

INT. DO NOT READ OUT, SPECIFY IT BASED ON THE RESPONDENT'S ANSWER. ONE ANSWER

1. Never
2. Very rarely (1-2 times)
3. Rarely (3-4 times)
4. Quite often (5-6 times)
5. Very often (over 6 times)
6. DO NOT READ OUT Not sure

BB4. Where will you most likely cycle if there are no bike paths and bike lanes?

INT. READ OUT. ONE ANSWER

1. On the sidewalk
2. On the roadway
3. Both options are acceptable
4. DO NOT READ OUT Not sure

AVAILABILITY

BC1. Please assess the sufficiency of bike paths and bike lanes in the city

Use 10-point scale, where 10 means “Fully sufficient”, 1 – “Not sufficient at all”.

INT. ONE ANSWER

Not sufficient at all									Fully sufficient
1	2	3	4	5	6	7	8	9	10

99 – not sure

BC2. How are you satisfied with the accessibility of your routes (no need to stop, carry a bicycle, walk, etc.)?

Use 10-point scale, where 10 means “Fully satisfied”, 1 – “Not satisfied at all”.

INT. ONE ANSWER

Not satisfied at all									Fully satisfied
1	2	3	4	5	6	7	8	9	10

99 – not sure

INTEGRITY

Let’s talk about the connection between cycling infrastructure and public transport.

BD1. Did you know that bicycles can be carried free of charge in urban land transport of any type?

INT. DO NOT READ OUT. ONE ANSWER

1. Yes
2. No

BD2. Do you combine cycling with public transport?

INT. READ OUT. ONE ANSWER

WHEN ANALIZING, LINK WITH THE AWARENESS (BD1)

1. No
2. I take my bike on public transit
3. I cycle to bus stops, public transport stations
4. DO NOT READ OUT. Not sure

ASK IF THERE IS CODE 2 IN BD2

BD3. What transport modes do you use to transport your bicycle?

INT. READ OUT. SEVERAL OPTIONS CAN BE CHOSEN

1. Land transport
2. MCC (Moscow Central Circle)
3. Commuter rail
4. Metro

BD4. How convenient is it to carry a bicycle on public transit?
Use 10-point scale, where 10 means "Very convenient", 1 – "Not convenient at all".
INT. ONE ANSWER
WHEN ANALYZING, STUDY IT BY TRANSPORT MODES

Question No.	Characteristic	Scale
4.1	Land transport (bus, trolleybus, tram)	1 2 3 4 5 6 7 8 9 10<br>99
4.2	MCC (Moscow Central Circle)	1 2 3 4 5 6 7 8 9 10<br>99
4.3	Commuter rail	1 2 3 4 5 6 7 8 9 10<br>99
4.4	Metro	1 2 3 4 5 6 7 8 9 10<br>99

CODES 2 OR 3 IN BA1
BD7. How satisfied are you with the number of bike rentals near public transport stations?
INT. READ OUT THE SCALE IF NECESSARY
Use 10-point scale, where 10 means "Fully satisfied", 1 – "Not satisfied at all".
INT. ONE ANSWER PER EACH LINE
IF A RESPONDENT IS NOT SURE ABOUT HIS OR HER ANSWER, STATE 99 – NOT SURE
ADD ROTATION

Question No.	Characteristic	Scale
7.1	Land transport (bus, trolleybus, tram)	1 2 3 4 5 6 7 8 9 10<br>99
7.2	MCC (Moscow Central Circle)	1 2 3 4 5 6 7 8 9 10<br>99
7.3	Commuter rail	1 2 3 4 5 6 7 8 9 10<br>99
7.4	Metro	1 2 3 4 5 6 7 8 9 10<br>99

ATTRACTION

Let's talk about quality of cycling infrastructure

BE1. How satisfied are you with…?

INT. READ OUT THE SCALE IF NECESSARY

Use 10-point scale, where 10 means "Fully satisfied", 1 – "Not satisfied at all".

INT. ONE ANSWER PER EACH LINE

IF A RESPONDENT IS NOT SURE ABOUT HIS OR HER ANSWER, STATE 99 – NOT SURE

ADD ROTATION

Question No.	Characteristic	Scale
1.1	Additional infrastructure on route (greenery, water kiosks, waste containers, toilets)	1 2 3 4 5 6 7 8 9 10 99
1.2	Lighting of bike paths	1 2 3 4 5 6 7 8 9 10 99
1.3	Traffic safety for you in parks and parkland	1 2 3 4 5 6 7 8 9 10 99
1.4	Traffic safety for you in not crowded zones (industrials zones, alleys, blind alleys)	1 2 3 4 5 6 7 8 9 10 99
1.5	Safekeeping of your bicycle near your house	1 2 3 4 5 6 7 8 9 10 99
1.6	Safekeeping of your bicycle in outdoor parking lots in the city	1 2 3 4 5 6 7 8 9 10 99

BE2. Do you use mobile apps to navigate or receive information on cycling?

INT. ONE ANSWER

1. Yes SPECIFY THE APPS _______
2. No

BE3. In your opinion, what could be added in navigation and cycling information apps?

WRITE IN THE ANSWER

____

____

99 – not sure

BE4. Do you have enough information on city cycling infrastructure to plan your bike rides?

INT. READ OUT THE SCALE IF NECESSARY

Use 10-point scale, where 10 means "Fully enough", a 1 – "Not enough at all".

INT. ONE ANSWER PER EACH LINE

IF A RESPONDENT IS NOT SURE ABOUT HIS OR HER ANSWER, STATE 99 – NOT SURE

ADD ROTATION

WHEN ANALYZING, LINK WITH THE USE OF BICYCLES (BA1 – OWN OR RENTAL BICYCLE)

Question No.	Characteristic	Scale
4.1	Information on bicycle parking	1 2 3 4 5 6 7 8 9 10<br>99
4.2	Information on bike rentals	1 2 3 4 5 6 7 8 9 10<br>99
4.3	Information on route distance (how long a ride will take)	1 2 3 4 5 6 7 8 9 10<br>99
4.4	Information on bike paths and bike lanes	1 2 3 4 5 6 7 8 9 10<br>99
4.5	Information on cycling traffic density on route	1 2 3 4 5 6 7 8 9 10<br>99

CONVENIENCE

Now, let's talk about convenience of cycling in Moscow

BF1. How satisfied are you with the following characteristics?

INT. READ OUT THE SCALE IF NECESSARY

Use 10-point scale, where 10 means "Fully satisfied", 1 – "Not satisfied at all".

INT. ONE ANSWER PER EACH LINE

IF A RESPONDENT IS NOT SURE ABOUT HIS OR HER ANSWER, STATE 99 – NOT SURE

ADD ROTATION

Question No.	Characteristic	Scale
1.1	Quality of bike path surface<br><br>*(READ OUT IF NECESSARY a bike path is a part of a shared-use road or a separate road dedicated to cycling)*	1 2 3 4 5 6 7 8 9 10<br>99
1.2	Quality of shared-use path surface for cyclists<br><br>*(READ OUT IF NECESSARY a shared-use path is a road element separated from a roadway (or a separate road) dedicated for cycling or both for cyclists and pedestrians)*	1 2 3 4 5 6 7 8 9 10<br>99
1.3	Quality of bike lane surface on roadways<br><br>*(READ OUT IF NECESSARY a bike lane is a part of a roadway, which is dedicated for cycling. Bike lanes are separated from the rest of roadways with lengthwise road surface marking)*	1 2 3 4 5 6 7 8 9 10<br>99

BF2. Would you like to use a bicycle as a mode of transportation instead of a car or public transport, or do it more often?

INT. READ OUT. ONE ANSWER

1. Yes
2. Yes, under certain conditions
3. No
4. DO NOT READ OUT. Not sure
5.

BF5. How satisfied are you with….

INT. READ OUT THE SCALE IF NECESSARY

Use 10-point scale, where 10 means "Fully satisfied", 1 – "Not satisfied at all".

INT. ONE ANSWER PER EACH LINE

IF A RESPONDENT IS NOT SURE ABOUT HIS OR HER ANSWER, STATE 99 – NOT SURE

ADD ROTATION

Question No.	Characteristic	Scale
5.1	Convenience of bike parking near your house	1 2 3 4 5 6 7 8 9 10 99
5.2	Bike storage near your house	1 2 3 4 5 6 7 8 9 10 99
5.3	Parking lots near the place where you work/study	1 2 3 4 5 6 7 8 9 10 99
5.4	Changing rooms at the place where you work/study	1 2 3 4 5 6 7 8 9 10 99
5.5	Possibility to have a shower after rides at the place where you work/study	1 2 3 4 5 6 7 8 9 10 99

BF6. If there were more convenient conditions for cyclists in your company / educational institution, would you cycle to work / educational institution or do it more often?

INT. READ OUT. SEVERAL OPTIONS

1. I do it already
2. Likely I would
3. Likely I wouldn't
4. DO NOT READ. Not sure

GO TO QUESTION A13

POTENTIAL USERS

ASK IF THERE IS CODE 2 IN A5

PA3. Which transport modes do you use to move around the city at least once a week?

INT. READ OUT, SEVERAL OPTIONS CAN BE CHOSEN

1. Urban public transport
2. Private car
3. Taxi
4. Motorcycle
5. Carsharing
6. Scooter, skateboard, etc.
7. Other ___________ INT. WRITE IN THE ANSWER

ASK IF THERE IS CODE 1 IN PA3

PA4. Which public transport modes do you use (at least once a week)?

INT. READ OUT, SEVERAL OPTIONS CAN BE CHOSEN

1. Land transport (bus, trolleybus, tram)
2. MCC (Moscow Central Circle)
3. Commuter rail
4. Metro

ASK IF THERE IS CODE 1 IN PA3

PA5 Where do you go by public transport more often?

INT. READ OUT, SEVERAL OPTIONS CAN BE CHOSEN

1. Work/educational institution and home
2. Leisure activity/meeting with friends
3. Shopping
4. Other activities not related to work
5. Sports or another hobby
6. Other ___________ INT. WRITE IN THE ANSWER

ASK IF THERE IS CODE 1 IN PA3

PA6. How long is your average trip on public transport?

INT. READ OUT. ONE ANSWER

1. Up to 15 min
2. 15-30 min
3. 30 min – 1 hour
4. Over 1 hour
5. DO NOT READ OUT Not sure/ No answer

ASK IF THERE IS CODE 1 IN PA3

PA7. How satisfied are you with moving around the city by public transport?

Use 10-point scale, where 10 means “Fully satisfied”, 1 – “Not satisfied at all”.

Transport mode	Scale
Land transport	1 2 3 4 5 6 7 8 9 10 99
MCC (Moscow Central Circle)	1 2 3 4 5 6 7 8 9 10 99
Commuter rail	1 2 3 4 5 6 7 8 9 10 99
Metro	1 2 3 4 5 6 7 8 9 10 99

ASK IF THERE IS CODE 2 IN PA3

PA8. Where do you go by car more often?

INT. READ OUT, SEVERAL OPTIONS CAN BE CHOSEN

1. Work/educational institution and home
2. Leisure activity/meeting with friends
3. Shopping
4. Other activities not related to work
5. Sports or another hobby
6. Other ___________ INT. WRITE IN THE ANSWER

ASK IF THERE IS CODE 2 IN PA3

PA10. How satisfied are you with moving around the city by car?

Use 10-point scale, where 10 means "Fully satisfied", 1 – "Not satisfied at all".

Not satisfied at all									Fully satisfied
1	2	3	4	5	6	7	8	9	10

99 – not sure

ASK IF THERE IS CODE 3 IN PA3

PA11 Where do you go by taxi more often?

INT. READ OUT, SEVERAL OPTIONS CAN BE CHOSEN

1. Work/educational institution and home
2. Leisure activity/meeting with friends
3. Shopping
4. Other activities not related to work
5. Sports or another hobby
6. Other __________ INT. WRITE IN THE ANSWER

ASK IF THERE IS CODE 4 IN PA3

PA14 Where do you go by motorcycle more often?

INT. READ OUT, SEVERAL OPTIONS CAN BE CHOSEN

1. Work/educational institution and home
2. Leisure activity/meeting with friends
3. Shopping
4. Other activities not related to work
5. Sports or another hobby
6. Other __________ INT. WRITE IN THE ANSWER

ASK IF THERE IS CODE 4 IN PA3

PA16. How satisfied are you with moving around the city by motorcycle?

Use 10-point scale, where 10 means "Fully satisfied", 1 – "Not satisfied at all".

Not satisfied at all									Fully satisfied
1	2	3	4	5	6	7	8	9	10

99 – not sure

ASK IF THERE IS CODE 5 IN PA3

PA17. Where do you go using carsharing more often?

INT. READ OUT, SEVERAL OPTIONS CAN BE CHOSEN

1. Work/educational institution and home
2. Leisure activity/meeting with friends
3. Shopping
4. Other activities not related to work
5. Sports or another hobby
6. Other __________ INT. WRITE IN THE ANSWER

ASK IF THERE IS CODE 5 IN PA3

PA19. How satisfied are you with using carsharing to move around the city?
Use 10-point scale, where 10 means "Fully satisfied", 1 – "Not satisfied at all".

Not satisfied at all									Fully satisfied
1	2	3	4	5	6	7	8	9	10

99 – not sure

PA20. In general, are you ready to move around the city by bike?
Use 10-point scale, where 10 means "Certainly ready", 1 – "Not ready at all".

Not ready at all									Certainly ready
1	2	3	4	5	6	7	8	9	10

99 – not sure

IF THERE ARE CODES 1-6 IN PA18 OR A8 (MOVING LESS OFTEN THAN ONCE A MONTH / NOT CYCLING AND NOT READY DO IT MORE OFTEN)

A12. What's the main reason why you don't use a bicycle as a transport mode instead of a car or public transport?
OPEN-ENDED QUESTION, WRITE THE RESPONDENT'S ANSWER

....

....

A12. What would encourage you to use a bicycle as a transport mode instead of a car or public transport?
OPEN-ENDED QUESTION, WRITE THE RESPONDENT'S ANSWER

DEVELOPMENT OF CYCLING INFRASTRUCTURE IN THE CITY

Finally, a few general questions.

ASK EVERYBODY (CYCLISTS AND POTENTIAL USERS, WHEN ANALYZING, DIVIDE THEM)

A13. In your opinion, does the development of cycling infrastructure in the city influence…

INT. READ OUT. SEVERAL OPTIONS
Use 10-point scale, where 10 means "Extremely slight impact", 1 – "Extremely significant impact".

Question number	Characteristic	Scale
P1.1	Megalopolis economy	1 2 3 4 5 6 7 8 9 10 99
P1.2	Citizens' health	1 2 3 4 5 6 7 8 9 10 99
P1.3	Environment in the city	1 2 3 4 5 6 7 8 9 10 99
P1.4	Transport conditions	1 2 3 4 5 6 7 8 9 10 99

A14. In your opinion, is it important to develop cycling infrastructure in Moscow?

INT. READ OUT THE SCALE IF NECESSARY
Use 10-point scale, where 10 means "Very important", 1 – "Not important at all".

Not important at all									Very important
1	2	3	4	5	6	7	8	9	10

A15. Assess the importance of the cycling infrastructure development:

INT. ONE ANSWER PER EACH LINE
IF A RESPONDENT IS NOT SURE ABOUT HIS OR HER ANSWER, STATE 99 – NOT SURE
ADD ROTATION

Question number	Characteristic	Scale
14.1	In Central Okrug	1 2 3 4 5 6 7 8 9 10 99
14.2	Between the Garden Ring and the Third Ring	1 2 3 4 5 6 7 8 9 10 99
14.3	In bedroom communities	1 2 3 4 5 6 7 8 9 10 99
14.4	Between residential areas and transport hubs (train stations, bus stations, airports, etc.)	1 2 3 4 5 6 7 8 9 10 99
14.5	In parkland	1 2 3 4 5 6 7 8 9 10 99

A16. Choose three areas of the cycling infrastructure development, which are the most important to you.

INT. READ OUT. UP TO 3 OPTIONS
OPTION ROTATION

1. The single connected network of bike paths in the city
2. Bike lanes and bike paths on the streets
3. Bike paths in parks
4. Bicycle parking lots near public buildings, shops, etc.
5. Special places to store bicycles near your house
6. Special places to store bicycles near transport hubs
7. Velobike Moscow Bikesharing
8. Possibility and convenience of bicycle transportation by bus, trolleybus, MCC and commuter rail
9. Creating comfortable conditions for cyclists at work or at educational institutions (shower, bike storage, etc.)
10. Maintenance of cycling infrastructure in the winter (e.g. snow removal on bike paths)
11. Dockless bicycle sharing
12. Cycling rules training
13. Bike parades and other events in the city
14. Other_______ INT. WRITE IN THE ANSWER
15. DO NOT READ OUT. Not sure

Thank you for your answers!
